# Conceptual DFT Descriptors of Amino Acids with Potential Corrosion Inhibition Properties Calculated with the Latest Minnesota Density Functionals

**DOI:** 10.3389/fchem.2017.00016

**Published:** 2017-03-16

**Authors:** Juan Frau, Daniel Glossman-Mitnik

**Affiliations:** ^1^Departament de Química, Universitat de les Illes BalearsPalma de Mallorca, Spain; ^2^Laboratorio Virtual NANOCOSMOS, Departamento de Medio Ambiente y Energía, Centro de Investigación en Materiales AvanzadosChihuahua, Mexico

**Keywords:** computational chemistry, conceptual DFT, Minnesota density functionals, amino acids, corrosion inhibition

## Abstract

Amino acids and peptides have the potential to perform as corrosion inhibitors. The chemical reactivity descriptors that arise from Conceptual DFT for the twenty natural amino acids have been calculated by using the latest Minnesota family of density functionals. In order to verify the validity of the calculation of the descriptors directly from the HOMO and LUMO, a comparison has been performed with those obtained through ΔSCF results. Moreover, the active sites for nucleophilic and electrophilic attacks have been identified through Fukui function indices, the dual descriptor Δf(**r**) and the electrophilic and nucleophilic Parr functions. The results could be of interest as a starting point for the study of large peptides where the calculation of the radical cation and anion of each system may be computationally harder and costly.

## 1. Introduction

Conceptual Density Functional Theory (DFT) or Chemical Reactivity Theory (as it is also known) is a powerful tool for the prediction, analysis and interpretation of the outcome of chemical reactions (Parr and Yang, 1989; Geerlings et al., 2003; Toro-Labbé, 2007; Chattaraj, 2009).

An interesting chemical reaction amenable of being studied through Conceptual DFT is the electron transfer between an electrodonating organic molecule and a metallic surface thus avoiding the corrosion (or oxidation) process. This constitutes the foundation of the theoretical studies of the molecular properties of corrosion inhibitors and there is a vast amount of scientific literature dedicated to it (Raja et al., 2016 and references herein).

Recently, a number of studies have been published about the possibility of use of natural amino acids as corrosion inhibitors (Fu et al., 2010; Dehdab et al., 2016; Kaya et al., 2016). Indeed, this idea could be extended to small peptides (Muruve et al., 2016). However, the problem of corrosion is pH-dependent. Therefore, the molecular structure of the amino acids being considered as potential corrosion inhibitors will be different according to the pH of the environment.

Therefore, we believe that it could be of interest to apply the concepts of Density Functional Theory to the study of natural amino acids bearing an ionizable side-chain (Arg, Asp, Glu, His, and Lys) in order to find if it is possible to discern between their potential as possible corrosion inhibitors.

Following the pioneering work of Parr and Yang (1989), an useful number of concepts have been derived from the analysis of the density of any molecular system through DFT. These concepts that allows a researcher to make qualitative predictions about the chemical reactivity of a given system, can also be quantified and are collectively known as Conceptual DFT Descriptors.

In order to obtain quantitative values of the Conceptual DFT Descriptors, it is necessary to resort to the Kohn-Sham theory trough calculations of the molecular density, the energy of the system, and the orbital energies, in particular, those related to the frontier orbitals, that is, HOMO and LUMO (Young, 2001; Lewars, 2003; Cramer, 2004; Jensen, 2007).

Although the foundations of DFT have established that an universal density functional must exist, and that all of the properties of the system can be obtained through calculations with this functional, in practice one needs to resort to some of the approximate density functionals that have been developed during the last 30 years. Due to the fact that these are approximate functionals (that is, not an universal functional), many of them are good for predicting some properties and others are good for another properties. Sometimes, you can find density functionals that are excellent for describing the properties of a given molecular system with a particular functional group, but it is necessary to resort to other density functionals for a different functional group that you want to include in the molecular system under study.

When one is dealing with the study of the chemical reactivity, that is, a process that involve the transference of electrons, it is usual to perform calculations not only of the ground state, but also for open systems like the radical cation and radical anion. These systems are often difficult to converge giving trustworthy results, specially if diffuse functions must be included in the basis set (Young, 2001; Lewars, 2003; Cramer, 2004; Jensen, 2007). For this reason, it is convenient to have a method that can give all information that one needs directly from the results of the calculation of the ground state of the molecular system under study. In particular, one may want to obtain the ionization potential (I) and electron affinity (A) of the system avoiding the calculation of the radicals anion and cation. Indeed, the link for this is given by the so-called Koopmans' theorem (Young, 2001; Lewars, 2003; Cramer, 2004; Jensen, 2007).

However, the Koopmans' theorem is not valid within DFT. Notwithstanding, from an empirical and practical point of view, it meaningful to follow the procedure of assigning the KS HOMO as equal to and opposite of the vertical ionization potential, ϵ_*H*_ = −I and the KS LUMO as equal to and opposite of the vertical electron affinity, ϵ_*L*_ = −A. We have coined the acronym KID for this empirical procedure (for “Koopmans in DFT”). For vertical ionization potential and vertical electron affinity we mean the differences between the energies of the radical cation and the neutral molecule and between the neutral molecule and anion radical respectively, all of them calculated at the geometry of the neutral. This is a necessary condition because the Conceptual DFT descriptors are defined and calculated at constant external potential v(**r**).

This means that the goodness of a given density functional for the purpose of predicting the Conceptual DFT descriptors directly from the properties of the neutral molecule can be estimated by checking how well it follows the KID procedure. Thus, it will be interesting to consider several recent density functionals that have shown great accuracy across a broad spectrum of databases in chemistry and physics (Peverati and Truhlar, 2014) to evaluate their performance in the fulfilling of this practical technique.

The objective of this work is twofold: (i) to conduct a comparative study of the performance of some density functionals from the Minnesota family for the description of the chemical reactivity of some natural amino acids bearing a ionizable side-chain (Arg, Asp, Glu, His, and Lys) ; and (ii) to analyze the potential of the studied amino acids to act as corrosion inhibitors on the basis of the calculated Conceptual DFT Descriptors.

**Figure 1:**
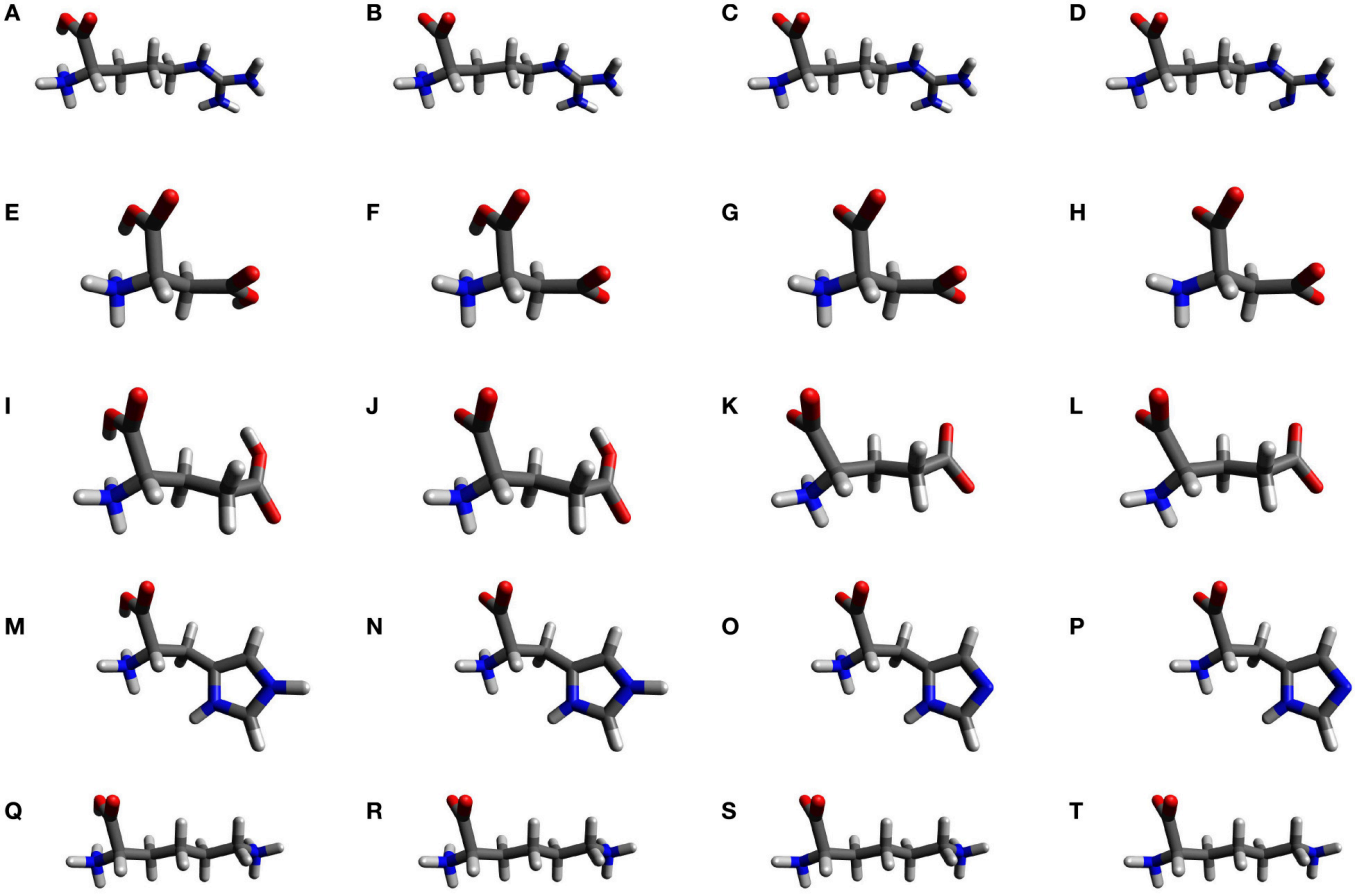
**The molecular structures of the natural amino acids bearing a ionizable side-chain (Arg, Asp, Glu, His, and Lys) at different pHs: (A)** Arg1, **(B)** Arg2, **(C)** Arg3, **(D)** Arg4, **(E)** Asp1, **(F)** Asp2, **(G)** Asp3, **(H)** Asp4, **(I)** Glu1, **(J)** Glu2, **(K)** Glu3, **(L)** Glu4, **(M)** His1, **(N)** His2, **(O)** His3, **(P)** His3, **(Q)** Lys1, **(R)** Lys2, **(S)** Lys3, and **(T)** Lys4.

As these amino acids present three ionization sites, there will be four structures for each molecule on the whole range of pH. We have labeled them with the indices 1–4, according to an increasing pH, and their structures are shown in Figure 1.

## 2. Theoretical background

As this work is part of an ongoing project, the theoretical background is similar to that presented in previous research (Glossman-Mitnik, 2013a,b, 2014a,b; Martínez-Araya et al., 2013a,b) and will be shown here for the sake of completeness. Within the conceptual framework of DFT (Parr and Yang, 1984; Geerlings et al., 2003), the chemical potential μ is defined as:

(1) $$\begin{array}{l} \mu ={\left(\frac{\partial E}{\partial N}\right)}_{v(\text{r})}=-\chi \end{array}$$

where χ is the electronegativity, while the global hardness η is:

(2) $$\begin{array}{l} \eta ={\left(\frac{\partial^{2}E}{\partial N^{2}}\right)}_{v\left(\text{r}\right)} \end{array}$$

Using a finite difference approximation and the KID procedure, the above expressions can be written as:

(3) $$\begin{array}{l} \mu =-\frac{1}{2}\left(I+A\right)\approx \frac{1}{2}\left(\epsilon_{L}+\epsilon_{H}\right)=\chi_{K} \end{array}$$

(4) $$\begin{array}{l} \eta =\left(I-A\right)\approx \left(\epsilon_{L}-\epsilon_{H}\right)=\eta_{K} \end{array}$$

where ϵ_*H*_ and ϵ_*L*_ are the energies of the highest occupied and the lowest unoccupied molecular orbitals, HOMO and LUMO, respectively. The electrophilicity index ω has been defined as Parr et al. (1999):

(5) $$\begin{array}{l} \omega =\frac{\mu^{2}}{2\eta }=\frac{{\left(I+A\right)}^{2}}{4\left(I-A\right)}\approx \frac{{\left(\epsilon_{L}+\epsilon_{H}\right)}^{2}}{4\left(\epsilon_{L}-\epsilon_{H}\right)}=\omega_{K} \end{array}$$

The electrodonating (ω^−^) and electroaccepting (ω^+^) powers have been defined as Gázquez et al. (2007):

(6) $$\begin{array}{l} \omega^{-}=\frac{{\left(3I+A\right)}^{2}}{16\left(I-A\right)}\approx \frac{{\left(3\epsilon_{H}+\epsilon_{L}\right)}^{2}}{16\eta_{K}}=\omega_{K}^{-} \end{array}$$

(7) $$\begin{array}{l} \omega^{+}=\frac{{\left(I+3A\right)}^{2}}{16\left(I-A\right)}\approx \frac{{\left(\epsilon_{H}+3\epsilon_{L}\right)}^{2}}{16\eta_{K}}=\omega_{K}^{+} \end{array}$$

It follows that a larger ω^+^ value corresponds to a better capability of accepting charge, whereas a smaller value of ω^−^ makes it a better electron donor. In order to compare ω^+^ with −ω^−^, the following definition of net electrophilicity has been proposed (Chattaraj et al., 2009):

(8) $$\begin{array}{l} \Delta \omega^{\pm }=\omega^{+}-\left(-\omega^{-}\right)=\omega^{+}+\omega^{-}\approx \omega_{K}^{+}-\left(-\omega_{K}^{-}\right)\\ =\omega_{K}^{+}+\omega_{K}^{-}=\Delta \omega_{K}^{\pm } \end{array}$$

that is, the electroaccepting power relative to the electrodonating power. The Fukui function is defined in terms of the derivative of ρ(**r**) with respect to *N* (Geerlings et al., 2003):

(9) $$\begin{array}{l} f\left(\text{r}\right)={\left(\frac{\partial \rho \left(\text{r}\right)}{\partial N}\right)}_{\upsilon \left(\text{r}\right)} \end{array}$$

The function *f*(**r**) reflects the ability of a molecular site to accept or donate electrons. High values of *f*(**r**) are related to a high reactivity at point **r** (Geerlings et al., 2003). By applying a finite difference approximation to Equation (9), two definitions of Fukui functions depending on total electronic densities are obtained:

(10) $$\begin{array}{lll} f^{+}\left(\text{r}\right) & = & \rho_{N+1}\left(\text{r}\right)-\rho_{N}\left(\text{r}\right) \end{array}$$

(11) $$\begin{array}{lll} f^{-}\left(\text{r}\right) & = & \rho_{N}\left(\text{r}\right)-\rho_{N-1}\left(\text{r}\right) \end{array}$$

where ρ_*N*+1_(**r**), ρ_*N*_(**r**) and ρ_*N*−1_(**r**) are the electronic densities at point **r** for the system with *N* + 1, *N* and *N* − 1 electrons, respectively. The first one, *f*^+^(**r**), has been associated to reactivity for a nucleophilic attack so that it measures the intramolecular reactivity at the site **r** toward a nucleophilic reagent. The second one, *f*^−^(**r**), has been associated to reactivity for an electrophilic attack so that this function measures the intramolecular reactivity at the site **r** toward an electrophilic reagent (Parr and Yang, 1984).

Morell et al. (Morell et al., 2005, 2006, 2008a,b; Ayers et al., 2007; Toro-Labbé, 2007; Cárdenas et al., 2009) have proposed a local reactivity descriptor (LRD) (that is, that allows to study the individual sites within the molecule rather than the global system) which is called the dual descriptor (DD) *f*^(2)^(**r**) ≡ Δ*f*(**r**). The definition of Δ*f*(**r**) is shown as indicated by Morell et al. (2005, 2006):

(12) $$\begin{array}{l} \Delta f\left(\text{r}\right)={\left(\frac{\partial f\left(\text{r}\right)}{\partial N}\right)}_{\upsilon \left(\text{r}\right)} \end{array}$$

The dual descriptor can be condensed over the atomic sites: When Δ*f*_*k*_ > 0 the process is driven by a nucleophilic attack on atom *k* and then that atom acts as an electrophilic species; conversely, when Δ*f*_*k*_ < 0 the process is driven by an electrophilic attack over atom *k* and therefore atom *k* acts as a nucleophilic species. In 2014, Domingo proposed the Parr functions P(**r**) (Chamorro et al., 2013; Domingo et al., 2013) which are given by the following equations:

(13) $$\begin{array}{l} P^{-}\left(\text{r}\right)=\rho_{s}^{rc}\left(\text{r}\right)\;\left(\text{for electrophilic attacks}\right) \end{array}$$

(14) $$\begin{array}{l} P^{+}\left(\text{r}\right)=\rho_{s}^{ra}\left(\text{r}\right)\;\left(\text{for nucleophilic attacks}\right) \end{array}$$

which are related to the atomic spin density (ASD) at the **r** atom of the radical cation or anion of a given molecule, respectively. The ASD over each atom of the radical cation and radical anion of the molecule gives the local nucleophilic ${\text{P}}_{k}^{-}$ and electrophilic ${\text{P}}_{k}^{+}$ Parr functions of the neutral molecule (Domingo et al., 2016).

## 3. Settings and computational methods

Following the lines of our previous work (Glossman-Mitnik, 2013a,b, 2014a,b; Martínez-Araya et al., 2013a,b), the computational studies were performed with the Gaussian 09 (Frisch et al., 2009) series of programs with density functional methods as implemented in the computational package. The force constants and vibrational frequencies were determined by computing analytical frequencies on the stationary points obtained after the optimization to check if there were true minima. The basis set used in this work was Def2SVP for geometry optimization and frequencies while Def2TZVP was considered for the calculation of the electronic properties (Weigend and Ahlrichs, 2005; Weigend, 2006).

For the calculation of the molecular structure and properties of the studied systems, we have chosen four density functionals from the latest Minnesota family, which consistently provide satisfactory results for several structural and thermodynamic properties (Peverati and Truhlar, 2014): M11L, which is a dual-range local meta-GGA (Peverati and Truhlar, 2012b), MN12L, which is a nonseparable local meta-NGA (Peverati and Truhlar, 2012a), MN12SX, which is a range-separated hybrid nonseparable meta-NGA (Peverati and Truhlar, 2012c) and N12SX, which is a range-separated hybrid nonseparable gradient approximation (Peverati and Truhlar, 2012c). In these functionals, GGA stands for generalized gradient approximation (in which the density functional depends on the up and down spin densities and their reduced gradient) and NGA stands for nonseparable gradient approximation (in which the density functional depends on the up/down spin densities and their reduced gradient, and also adopts a nonseparable form). All the calculations were performed in the presence of water as a solvent, by doing IEF-PCM computations according to the SMD solvation model (Marenich et al., 2009).

## 4. Results and discussion

The molecular structures of the natural amino acids bearing a ionizable side-chain (Arg, Asp, Glu, His, and Lys) at different pHs were pre-optimized by starting with the readily available MOL structures (ChemSpider: www.chemspider.com, PubChem: https://pubchem.ncbi.nlm.nih.gov/), and finding the most stable conformers by means of the Avogadro 1.2.0 program (Hanwell et al., 2012) through a random sampling with molecular mechanics techniques and a consideration of all the torsional angles through the general AMBER force field (Wang et al., 2004). The structures of the resulting conformers were then reoptimized with the four density functionals mentioned in the previous section in conjunction with the Def2SVP basis set and the SMD solvation model, using water as a solvent.

In order to check for the applicability of the KID procedure, it is worth to calculate the electronegativity χ, the global hardness η and the global electrophilicity ω for the studied systems using both approximations in order to verify the agreement with the vertical ΔSCF derived values. Additionally, we will include in the calculations, the electrodonating (ω^−^) and electroaccepting (ω^+^) powers as well as the net electrophilicity Δω^±^ for further verifications.

The HOMO and LUMO orbital energies (in eV), ionization potentials I and electron affinities A (in eV), and global electronegativity χ, total hardness η, global electrophilicity ω, electrodonating power, (ω^−^), electroaccepting power (ω^+^), and net electrophilicity Δω^±^ of the twenty molecular structures calculated with the M11L, MN12L, MN12SX, and N12SX density functionals and the Def2TZVP basis set using water as solvent simulated with the SMD parametrization of the IEF-PCM model are presented in Tables S1A–S4A of the Electronic Supplementary Materials (ESM). The upper part of the tables shows the results derived assuming the validity of the KID procedure (hence the subscript K) and the lower part shows the results derived from the calculated vertical ΔSCF energies.

We have previously designed several descriptors that relate the results obtained through the HOMO and LUMO calculations with those obtained by means of the vertical I and A with a ΔSCF procedure. However, it must be stressed that it is not our intention to perform a gap-fitting by minimizing a descriptor by choosing optimal range-separation parameter γ, but to check if the density functionals considered in this study, in which, some of them contain a fixed range-separation parameter γ, follow the KID procedure. As a matter fact, there is no range-separation parameter γ in our designed descriptors. Moreover, we have considered A as minus the energy of the LUMO of the neutral system instead of considering A as minus the energy of the HOMO of the N+1 electron system, as it was in some recent works (Kronik et al., 2012; Lima et al., 2016).

The first three descriptors are related to the simplest fulfillment of the KID procedure by relating ϵ_*H*_ with −I, ϵ_*L*_ with −A, and the behavior of them in the description of the HOMO-LUMO gap: *J*_*I*_ = |ϵ_*H*_ + *E*_*gs*_(*N* − 1) − *E*_*gs*_(*N*)|, *J*_*A*_ = |ϵ_*L*_ + *E*_*gs*_(*N*)−*E*_*gs*_(*N* + 1)| and $J_{HL}=\sqrt{{J_{I}}^{2}+{J_{A}}^{2}}$.

Next, we consider four other descriptors that analyze how well the studied density functionals are useful for the prediction of the electronegativity χ, the global hardness η and the global electrophilicity ω, and for a combination of these Conceptual DFT descriptors, just considering the energies of the HOMO and LUMO or the vertical I and A: *J*_χ_ = |χ − χ_*K*_|, *J*_η_ = |η − η_*K*_|, *J*_ω_ = |ω − ω_*K*_| and $J_{D1}=\sqrt{J_{\chi }^{2}+J_{\eta }^{2}+J_{\omega }^{2}}$, where D1 stands for the first group of Conceptual DFT descriptors.

Finally, we designed other four descriptors to verify the goodness of the studied density functionals for the prediction of the electrodonating power ω^−^, the electroaccepting power ω^+^, the net electrophilicity Δω^±^ , and for a combination of these Conceptual DFT descriptors, just considering the energies of the HOMO and LUMO or the vertical I and A: $J_{\omega^{-}}=|\omega^{-}-\omega_{K}^{-}|$, $J_{\omega^{+}}=|\omega^{+}-\omega_{K}^{+}|$, $J_{\Delta \omega^{\pm }}=|\Delta \omega^{\pm }-\Delta \omega_{K}^{\pm }|$ and $J_{D2}=\sqrt{J_{\omega^{-}}^{2}+J_{\omega^{+}}^{2}+J_{\Delta \omega^{\pm }}^{2}}$, where D2 stands for the first group of Conceptual DFT descriptors.

The results of the calculations of J_*I*_, J_*A*_, J_*HL*_, J_χ_, J_η_, J_ω_, J_*D*1_, ${\text{J}}_{\omega^{-}}$, ${\text{J}}_{\omega^{+}}$, ${\text{J}}_{\Delta \omega^{\pm }}$, and J_*D*2_ for the natural amino acids bearing a ionizable side-chain at different pHs considered in this work are displayed in Tables S1B–S4B of the Electronic Supplementary Materials (ESM).

On the basis of the results for the descriptors presented on Tables S1B–S4B of the ESM, we have compiled the average values for for each density functional on the whole group of hexoses and pentoses, and the calculated results are displayed on Table 1.

**Table 1:** **Average descriptors J_***I***_, J_***A***_, J_***HL***_, J_**χ**_, J_**η**_, J_**ω**_, J_***D***1_, ${\text{J}}_{\omega^{-}}$, ${\text{J}}_{\omega^{+}}$, ${\text{J}}_{\Delta \omega^{\pm }}$, and J_***D*****2**_ for the natural amino acids bearing a ionizable side-chain at different pHs calculated from the results of Tables S1B–S4B of the ESM**.

	**J_*I*_**	**J_*A*_**	**J_*HL*_**	**J_χ_**	**J_η_**	**J_ω_**	**J_D1_**	**${\text{J}}_{\omega^{-}}$**	**${\text{J}}_{\omega^{+}}$**	**${\text{J}}_{\Delta \omega^{\pm }}$**	**J_D2_**
M11L	0.33	0.13	0.37	0.13	0.43	0.07	0.46	0.16	0.11	0.26	0.33
MN12L	0.26	0.22	0.35	0.12	0.36	0.09	0.43	0.22	0.12	0.34	0.43
MN12SX	0.24	0.41	0.52	0.32	0.33	0.18	0.51	0.51	0.20	0.71	0.90
N12SX	0.08	0.14	0.17	0.05	0.21	0.04	0.22	0.08	0.05	0.13	0.16

As can be seen from Table 1, the KID procedure holds with great accuracy for the N12SX density functionals, which is a range-separated hybrid NGA density functionals. Indeed, the values of J_*I*_, J_*A*_ and J_*HL*_ are not exactly zero. It is interesting to see that the same density functional also fulfill the KID procedure for the other descriptors, namely J_χ_, J_η_, J_ω_, and J_*D*1_. These results are very important, because they show that it is not enough to rely only in J_*I*_, J_*A*_, and J_*HL*_.

In spite of these results, a closer look at Tables S1A–S4A of the ESM reveals that all the density functionals considered in this work describe inadequately the energy of the LUMO for the molecular species at neutral or basic pH, leading to negative (or unphysical) values of the electron affinity A. For this reason, it is better to build a new table for the comparison of the average descriptors, but only considering the results at acid pH. In particular, we will consider Arg1, Asp1, Asp2, Glu1, Glu2, His1, and Lys1:

The results from Table 2 are very impressive. It is not only that the N12SX density functional is the best fulfilling the validity of the KID procedure, but also that the values are very close to zero. Therefore, from this observation one should be very careful in choosing the proper density functional for predictions in terms of the HOMO and LUMO energies, and that for the particular case of the study of corrosion inhibitors, this tool is only valid at acid pH.

**Table 2:** **Average descriptors J_***I***_, J_***A***_, J_***HL***_, J_**χ**_, J_**η**_, J_**ω**_, J_***D*****1**_, ${\text{J}}_{\omega^{-}}$, ${\text{J}}_{\omega^{+}}$, ${\text{J}}_{\Delta \omega^{\pm }}$, and J_***D*****2**_ for the natural amino acids bearing a ionizable side-chain at acid pH calculated from the results of Tables S1B–S4B of the ESM**.

	**J_*I*_**	**J_*A*_**	**J_*HL*_**	**J_χ_**	**J_η_**	**J_ω_**	**J_*D*1_**	**${\text{J}}_{\omega^{-}}$**	**${\text{J}}_{\omega^{+}}$**	**${\text{J}}_{\Delta \omega^{\pm }}$**	**J_*D*2_**
M11L	0.38	0.21	0.44	0.08	0.60	0.09	0.61	0.11	0.19	0.30	0.37
MN12L	0.30	0.33	0.44	0.04	0.62	0.14	0.64	0.25	0.23	0.48	0.58
MN12SX	0.28	0.07	0.29	0.17	0.21	0.07	0.29	0.24	0.07	0.30	0.39
N12SX	0.08	0.06	0.11	0.03	0.14	0.02	0.14	0.04	0.04	0.07	0.09

It is a common practice to compare the compare the corrosion inhibitor ability for a set of molecules in terms of their chemical hardness η. However, another possible comparison could be performed in terms of the electrodonating power ω^−^. By considering the values presented in Table S4A, the following trend can be established:

$$\begin{array}{l} \text{Asp}1\approx \text{Lys}1>\text{Glu}1>\text{His}1>\text{Arg}1>\text{Glu}2>\text{Asp}2 \end{array}$$

It is possible to evaluate condensed Fukui functions from single-points calculations directly, without resorting to additional calculations involving the systems with N − 1 and N + 1 electrons:

(15) $$f_{k^{+}}=\sum_{a\in k}\left[c_{ai^{2}}+c_{ai}\sum_{b\neq a}c_{bi}S_{ab}\right]\;(\text{where i = LUMO})$$

(16) $$f_{k^{-}}=\sum_{a\in k}\left[c_{ai^{2}}+c_{ai}\sum_{b\neq a}c_{bi}S_{ab}\right]\;(\text{where i = HOMO})$$

with c_*ai*_ being the LCAO coefficients and S_*ab*_ the overlap matrix. The condensed Fukui functions are normalized, thus $\underset{k}{\sum }f_{k}=1$ and $f_{k}^{0}$ = [$f_{k}^{+}+f_{k}^{-}$]/2.

The nucleophilic Fukui function ${\text{f}}_{k}^{-}$, the condensed dual descriptor Δf_*k*_ and the nucleophilic Parr function *P*^−^(**r**) over the carboxyl O atoms of the Asp1, Asp2, Glu1, Glu2, and Lys1 molecules calculated with the MN12SX and N12SX density functionals and the Def2TZVP basis set using water as solvent simulated with the SMD parametrization of the IEF-PCM model are shown in Table 3. For the calculation of the ASD, we have considered both a Mulliken Population Analysis (MPA) (Young, 2001; Lewars, 2003; Cramer, 2004; Jensen, 2007) or a Hirshfeld Population Analysis (HSA) (Hirshfeld, 1977; Ritchie, 1985; Ritchie and Bachrach, 1987) modified to render CM5 atomic charges (Marenich et al., 2012). The condensed Fukui functions and condensed dual descriptors have been calculated using the AOMix molecular analysis program (Gorelsky and Lever, 2001; Gorelsky, 2011) starting from single-point energy calculations.

**Table 3:** **Nucleophilic Fukui functions, condensed dual descriptors and nucleophilic Parr functions for the Asp1, Asp2, Glu1, Glu2, and Lys1 molecules calculated with the MN12SX and N12SX density functionals and the Def2TZVP basis set using water as solvent simulated with the SMD parametrization of the IEF-PCM model**.

	**MN12SX**				**N12SX**			
	**${\text{f}}_{\text{k}}^{-}$**	**Δf_k_**	**${\text{P}}_{k}^{-}$(MPA)**	**${\text{P}}_{\text{k}}^{-}$(HPA)**	**${\text{f}}_{\text{k}}^{-}$**	**Δf_k_**	**${\text{P}}_{\text{k}}^{-}$(MPA)**	**${\text{P}}_{k}^{-}$(HPA)**
Asp1	0.60	−0.60	0.42	0.37	0.66	−0.65	0.82	0.76
Asp2	0.75	−0.75	0.89	0.81	0.60	−0.60	0.86	0.80
Glu1	0.62	−0.62	0.85	0.77	0.67	−0.66	0.82	0.75
Glu2	0.74	−0.74	0.88	0.81	0.56	−0.56	0.57	0.51
Lys1	0.62	−0.52	0.84	0.76	0.66	−0.44	0.80	0.74

*MPA, Mulliken Population Analysis; HPA, Hirshfeld Population Analysis*.

For the case of His1, the nucleophilic Fukui function ${\text{f}}_{k}^{-}$, the condensed dual descriptor Δf_*k*_ and the nucleophilic Parr function *P*^−^(**r**) are delocalized over the C atoms of the imidazole side-chain group, while for Arg1 the descriptors are delocalized over the guanidinium side-chain group.

## 5. Conclusions

Some density functionals of the Minnesota family (M11L, MN12L, MN12SX, and N12SX) have been tested for the fulfillment of the KID procedure by comparison of the HOMO- and LUMO-derived values with those obtained through a ΔSCF procedure. The range-separated hybrid meta-NGA density functional (MN12SX) and the range-separated hybrid NGA density functional (N12SX) are the best for the accomplishment of this objective. As such, they represent a good prospect for their usefulness in the description of the chemical reactivity of molecular systems of large size.

From the observation of the whole of the results presented in this work, ione should be very careful in choosing the proper density functional for predictions in terms of the HOMO and LUMO energies, and that for the particular case of the study of corrosion inhibitors, this tool is only valid at acid pH. The amino acids with a COO^−^ side-chain group (Asp and Glu), together with Lys are the best candidates for the design of small peptides with potential to perform as corrosion inhibitors.

## Author contributions

DG conceived and designed the research and headed, wrote and revised the manuscript, while JF contributed to the writing and the revision of the article.

### Conflict of interest statement

The authors declare that the research was conducted in the absence of any commercial or financial relationships that could be construed as a potential conflict of interest. The reviewer MK and handling Editor declared their shared affiliation, and the handling Editor states that the process nevertheless met the standards of a fair and objective review.
