# Supplementary material for: Conceptual DFT Descriptors of Amino Acids with Potential Corrosion Inhibition Properties Calculated with the Latest Minnesota Density Functionals
Source: Front Chem. 2017 Mar 16;5:16. doi: 10.3389/fchem.2017.00016 (PMC5352669; doi:10.3389/fchem.2017.00016)
Supplement: Supplementary file 1 [file Table1.PDF]

Supplementary Material: Conceptual DFT Descriptors of Amino Acids with Potential Corrosion Inhibition Properties Calculated with the Latest Minnesota Density Functionals

Juan Frau and Daniel Glossman-Mitnik

*Correspondence:
Dr. Daniel Glossman-Mitnik
daniel.glossman@cimav.edu.mx

Table S1A. HOMO and LUMO orbital energies (eV), ionization potential I and electron affinity A (eV), global electronegativity χ, chemical hardness η, global electrophilicity ω, electrodonating power ω^-, electroaccepting power ω^+ and net electrophilicity $\Delta\omega^\pm$ of natural amino acids bearing a ionizable side-chain at different pHs calculated with the M11L density functional and the Def2TZVP basis set using water as solvent simulated with the SMD parametrization of the IEF-PCM model. The upper part of the table shows the results derived assuming the validity of the KID procedure and the lower part shows the results derived from the calculated vertical ΔSCF energies.

	HOMO	LUMO	χ_K	η_K	ω_K	ω_K^-	ω_K^+	$\Delta\omega_K^\pm$
Arg1	-6.959	-1.421	4.190	5.538	1.585	5.611	1.421	7.032
Arg2	-6.489	-0.403	3.446	6.086	0.976	4.055	0.609	4.664
Arg3	-6.003	-0.339	3.171	5.664	0.887	3.714	0.544	4.258
Arg4	-5.850	0.065	2.893	5.915	0.707	3.231	0.338	3.569
Asp1	-7.712	-1.390	4.551	6.322	1.638	5.946	1.395	7.342
Asp2	-6.403	-1.119	3.761	5.284	1.339	4.889	1.127	6.016
Asp3	-6.084	-0.401	3.243	5.683	0.925	3.827	0.584	4.411
Asp4	-5.612	0.115	2.748	5.727	0.659	3.051	0.303	3.354
Glu1	-7.397	-1.395	4.396	6.003	1.610	5.793	1.397	7.190
Glu2	-6.146	-1.362	3.754	4.785	1.473	5.121	1.367	6.489
Glu3	-6.039	-0.256	3.148	5.784	0.856	3.648	0.501	4.149
Glu4	-5.885	0.228	2.828	6.113	0.654	3.105	0.276	3.381
His1	-6.977	-1.523	4.250	5.454	1.656	5.778	1.528	7.305
His2	-6.426	-1.413	3.920	5.013	1.532	5.338	1.418	6.756
His3	-5.685	-0.444	3.065	5.241	0.896	3.652	0.587	4.239
His4	-5.354	-0.143	2.748	5.211	0.725	3.149	0.401	3.550
Lys1	-7.726	-1.369	4.547	6.357	1.626	5.924	1.376	7.300
Lys2	-6.472	-0.627	3.550	5.845	1.078	4.296	0.746	5.043
Lys3	-5.991	-0.594	3.293	5.397	1.004	3.993	0.700	4.693
Lys4	-5.952	0.117	2.918	6.069	0.701	3.241	0.323	3.564
	I	A	χ	η	ω	ω^-	ω^+	$\Delta\omega^\pm$
Arg1	7.449	1.189	4.319	6.260	1.490	5.531	1.212	6.742
Arg2	6.831	0.374	3.602	6.458	1.005	4.214	0.612	4.826
Arg3	6.365	0.319	3.342	6.046	0.924	3.896	0.554	4.450
Arg4	6.093	-0.023	3.035	6.115	0.753	3.406	0.371	3.777
Asp1	8.106	1.189	4.648	6.917	1.561	5.879	1.231	7.110
Asp2	6.713	0.975	3.844	5.738	1.288	4.856	1.012	5.867
Asp3	6.477	0.394	3.436	6.083	0.970	4.039	0.603	4.642
Asp4	5.927	-0.037	2.945	5.965	0.727	3.300	0.354	3.654
Glu1	7.779	1.161	4.470	6.618	1.509	5.667	1.198	6.865
Glu2	6.461	1.131	3.796	5.330	1.352	4.934	1.138	6.072
Glu3	6.342	0.268	3.305	6.073	0.899	3.831	0.526	4.356
Glu4	6.125	-0.142	2.992	6.267	0.714	3.316	0.324	3.640
His1	7.304	1.282	4.293	6.021	1.530	5.584	1.291	6.874
His2	6.722	0.985	3.853	5.736	1.294	4.874	1.020	5.894
His3	5.962	0.395	3.179	5.566	0.908	3.752	0.574	4.326
His4	5.616	0.199	2.907	5.416	0.780	3.353	0.446	3.799
Lys1	8.165	1.149	4.657	7.015	1.546	5.858	1.201	7.060
Lys2	6.812	0.522	3.667	6.290	1.069	4.365	0.698	5.063
Lys3	6.351	0.494	3.422	5.856	1.000	4.077	0.655	4.732
Lys4	6.233	-0.083	3.075	6.316	0.749	3.430	0.355	3.784

Table S1B. Descriptors J_I, J_A, J_{HL}, J_χ, J_η, J_ω, J_{D1}, J_{ω^-}, J_{ω^+}, $J_{\Delta\omega^\pm}$ and J_{D2} for the natural amino acids bearing a ionizable side-chain at different pHs calculated from the results of Table S1A

	J_I	J_A	J_{HL}	J_χ	J_η	J_ω	J_{D1}	J_{ω^-}	J_{ω^+}	$J_{\Delta\omega^\pm}$	J_{D2}
Arg1	0.49	0.23	0.54	0.13	0.72	0.09	0.74	0.08	0.21	0.29	0.37
Arg2	0.34	0.03	0.34	0.16	0.37	0.03	0.40	0.16	0.00	0.16	0.23
Arg3	0.36	0.02	0.36	0.17	0.38	0.04	0.42	0.18	0.01	0.19	0.26
Arg4	0.24	0.04	0.25	0.14	0.20	0.05	0.25	0.18	0.03	0.21	0.27
Asp1	0.39	0.20	0.44	0.10	0.60	0.08	0.61	0.07	0.16	0.23	0.29
Asp2	0.31	0.14	0.34	0.08	0.45	0.05	0.46	0.03	0.12	0.15	0.19
Asp3	0.39	0.01	0.39	0.19	0.40	0.05	0.45	0.21	0.02	0.23	0.31
Asp4	0.32	0.08	0.33	0.20	0.24	0.07	0.32	0.25	0.05	0.30	0.39
Glu1	0.38	0.23	0.45	0.07	0.62	0.10	0.63	0.13	0.20	0.32	0.40
Glu2	0.31	0.23	0.39	0.04	0.55	0.12	0.56	0.19	0.23	0.42	0.51
Glu3	0.30	0.01	0.30	0.16	0.29	0.04	0.33	0.18	0.02	0.21	0.28
Glu4	0.24	0.09	0.26	0.16	0.15	0.06	0.23	0.21	0.05	0.26	0.34
His1	0.33	0.24	0.41	0.04	0.57	0.13	0.58	0.19	0.24	0.43	0.53
His2	0.30	0.43	0.52	0.07	0.72	0.24	0.76	0.46	0.40	0.86	1.06
His3	0.28	0.05	0.28	0.11	0.32	0.01	0.34	0.10	0.01	0.09	0.13
His4	0.26	0.06	0.27	0.16	0.21	0.06	0.27	0.20	0.04	0.25	0.32
Lys1	0.44	0.22	0.49	0.11	0.66	0.08	0.67	0.07	0.18	0.24	0.30
Lys2	0.34	0.11	0.36	0.12	0.45	0.01	0.46	0.07	0.05	0.02	0.09
Lys3	0.36	0.10	0.37	0.13	0.46	0.00	0.48	0.08	0.05	0.04	0.10
Lys4	0.28	0.03	0.28	0.16	0.25	0.05	0.30	0.19	0.03	0.22	0.29
Average	0.33	0.13	0.37	0.13	0.43	0.07	0.46	0.16	0.11	0.26	0.33

Table S2A. HOMO and LUMO orbital energies (eV), ionization potential I and electron affinity A (eV), global electronegativity χ, chemical hardness η, global electrophilicity ω, electrodonating power ω^-, electroaccepting power ω^+ and net electrophilicity $\Delta\omega^\pm$ of natural amino acids bearing a ionizable side-chain at different pHs calculated with the MN12L density functional and the Def2TZVP basis set using water as solvent simulated with the SMD parametrization of the IEF-PCM model. The upper part of the table shows the results derived assuming the validity of the KID procedure and the lower part shows the results derived from the calculated vertical ΔSCF energies.

	HOMO	LUMO	χ_K	η_K	ω_K	ω_K^-	ω_K^+	$\Delta\omega_K^\pm$
Arg1	-6.875	-1.072	3.974	5.803	1.361	5.071	1.097	6.168
Arg2	-6.384	0.193	3.096	6.578	0.728	3.416	0.320	3.736
Arg3	-5.923	0.285	2.819	6.208	0.640	3.078	0.259	3.336
Arg4	-5.658	0.741	2.458	6.398	0.472	2.574	0.115	2.689
Asp1	-7.651	-1.115	4.383	6.537	1.469	5.539	1.156	6.695
Asp2	-6.262	-0.791	3.527	5.471	1.137	4.379	0.852	5.231
Asp3	-5.959	0.116	2.921	6.074	0.703	3.245	0.324	3.569
Asp4	-5.517	0.693	2.412	6.211	0.468	2.531	0.119	2.650
Glu1	-7.311	-1.083	4.197	6.228	1.414	5.316	1.119	6.435
Glu2	-6.005	-0.984	3.495	5.021	1.216	4.493	0.999	5.492
Glu3	-5.908	0.269	2.820	6.177	0.644	3.083	0.263	3.347
Glu4	-5.757	0.899	2.429	6.655	0.443	2.517	0.088	2.605
His1	-6.835	-1.188	4.011	5.647	1.425	5.208	1.197	6.405
His2	-6.197	-0.945	3.571	5.252	1.214	4.541	0.970	5.511
His3	-5.522	0.077	2.722	5.599	0.662	3.035	0.312	3.347
His4	-5.173	0.398	2.387	5.571	0.511	2.565	0.178	2.742
Lys1	-7.655	-1.064	4.359	6.591	1.442	5.475	1.115	6.590
Lys2	-6.374	-0.083	3.228	6.291	0.828	3.664	0.436	4.100
Lys3	-5.929	-0.050	2.989	5.879	0.760	3.382	0.393	3.774
Lys4	-5.889	0.742	2.573	6.631	0.499	2.700	0.126	2.826
	I	A	χ	η	ω	ω^-	ω^+	$\Delta\omega^\pm$
Arg1	7.271	0.725	3.998	6.546	1.221	4.850	0.852	5.702
Arg2	6.598	-0.138	3.230	6.736	0.774	3.585	0.355	3.940
Arg3	6.129	-0.307	2.911	6.436	0.658	3.174	0.263	3.437
Arg4	5.891	-0.475	2.708	6.366	0.576	2.903	0.196	3.099
Asp1	7.938	0.754	4.346	7.184	1.314	5.251	0.905	6.156
Asp2	6.466	0.454	3.460	6.012	0.996	4.097	0.637	4.735
Asp3	6.245	-0.068	3.089	6.312	0.756	3.450	0.361	3.812
Asp4	5.763	-0.456	2.653	6.219	0.566	2.848	0.194	3.042
Glu1	7.587	0.773	4.180	6.814	1.282	5.080	0.900	5.980
Glu2	6.209	0.636	3.423	5.572	1.051	4.162	0.739	4.901
Glu3	6.104	-0.204	2.950	6.308	0.690	3.248	0.299	3.547
Glu4	5.912	-0.550	2.681	6.461	0.556	2.857	0.176	3.032
His1	7.220	0.949	4.084	6.271	1.330	5.095	1.010	6.105
His2	6.422	0.619	3.520	5.803	1.068	4.258	0.738	4.997
His3	5.825	-0.152	2.837	5.976	0.673	3.138	0.302	3.440
His4	5.437	-0.240	2.599	5.677	0.595	2.844	0.245	3.089
Lys1	7.973	0.714	4.344	7.259	1.300	5.225	0.881	6.106
Lys2	6.581	-0.014	3.284	6.595	0.818	3.689	0.405	4.094
Lys3	6.262	-0.040	3.111	6.302	0.768	3.486	0.374	3.860
Lys4	6.072	-0.499	2.787	6.571	0.591	2.986	0.199	3.185

Table S2B. Descriptors J_I, J_A, J_{HL}, J_χ, J_η, J_ω, J_{D1}, J_{ω^-}, J_{ω^+}, $J_{\Delta\omega^\pm}$ and J_{D2} for the natural amino acids bearing a ionizable side-chain at different pHs calculated from the results of Table S2A

	J_I	J_A	J_{HL}	J_χ	J_η	J_ω	J_{D1}	J_{ω^-}	J_{ω^+}	$J_{\Delta\omega^\pm}$	J_{D2}
Arg1	0.40	0.35	0.53	0.02	0.74	0.14	0.76	0.22	0.25	0.47	0.57
Arg2	0.21	0.06	0.22	0.13	0.16	0.05	0.21	0.17	0.03	0.20	0.27
Arg3	0.21	0.02	0.21	0.09	0.23	0.02	0.25	0.10	0.00	0.10	0.14
Arg4	0.23	0.27	0.35	0.25	0.03	0.10	0.27	0.33	0.08	0.41	0.53
Asp1	0.29	0.36	0.46	0.04	0.65	0.15	0.67	0.29	0.25	0.54	0.66
Asp2	0.20	0.34	0.39	0.07	0.54	0.14	0.56	0.28	0.21	0.50	0.61
Asp3	0.29	0.05	0.29	0.17	0.24	0.05	0.30	0.20	0.04	0.24	0.32
Asp4	0.25	0.24	0.34	0.24	0.01	0.10	0.26	0.32	0.08	0.39	0.51
Glu1	0.28	0.31	0.41	0.02	0.59	0.13	0.60	0.24	0.22	0.45	0.56
Glu2	0.20	0.35	0.40	0.07	0.55	0.16	0.58	0.33	0.26	0.59	0.73
Glu3	0.20	0.06	0.21	0.13	0.13	0.05	0.19	0.17	0.04	0.20	0.26
Glu4	0.15	0.35	0.38	0.25	0.19	0.11	0.34	0.34	0.09	0.43	0.55
His1	0.39	0.24	0.45	0.07	0.62	0.09	0.64	0.11	0.19	0.30	0.37
His2	0.23	0.33	0.40	0.05	0.55	0.15	0.57	0.28	0.23	0.51	0.63
His3	0.30	0.07	0.31	0.11	0.38	0.01	0.39	0.10	0.01	0.09	0.14
His4	0.26	0.16	0.31	0.21	0.11	0.08	0.25	0.28	0.07	0.35	0.45
Lys1	0.32	0.35	0.47	0.02	0.67	0.14	0.68	0.25	0.23	0.48	0.59
Lys2	0.21	0.10	0.23	0.06	0.30	0.01	0.31	0.03	0.03	0.01	0.04
Lys3	0.33	0.09	0.35	0.12	0.42	0.01	0.44	0.10	0.02	0.09	0.14
Lys4	0.18	0.24	0.31	0.21	0.06	0.09	0.24	0.29	0.07	0.36	0.47
Average	0.26	0.22	0.35	0.12	0.36	0.09	0.43	0.22	0.12	0.34	0.43

Table S3A. HOMO and LUMO orbital energies (eV), ionization potential I and electron affinity A (eV), global electronegativity χ, chemical hardness η, global electrophilicity ω, electrodonating power ω^-, electroaccepting power ω^+ and net electrophilicity $\Delta\omega^\pm$ of natural amino acids bearing a ionizable side-chain at different pHs calculated with the MN12SX density functional and the Def2TZVP basis set using water as solvent simulated with the SMD parametrization of the IEF-PCM model. The upper part of the table shows the results derived assuming the validity of the KID procedure and the lower part shows the results derived from the calculated vertical ΔSCF energies.

	HOMO	LUMO	χ_K	η_K	ω_K	ω_K^-	ω_K^+	$\Delta\omega_K^\pm$
Arg1	-7.554	-1.079	4.316	6.475	1.439	5.440	1.124	6.564
Arg2	-7.289	-0.429	3.859	6.860	1.086	4.529	0.670	5.200
Arg3	-6.778	-0.383	3.581	6.395	1.002	4.195	0.614	4.809
Arg4	-6.347	0.119	3.114	6.466	0.750	3.461	0.347	3.807
Asp1	-8.591	-1.108	4.849	7.483	1.571	6.035	1.186	7.220
Asp2	-7.257	-0.924	4.090	6.333	1.321	5.083	0.992	6.075
Asp3	-6.981	-0.422	3.702	6.559	1.045	4.350	0.648	4.998
Asp4	-6.346	0.296	3.025	6.642	0.689	3.305	0.280	3.586
Glu1	-8.267	-1.060	4.664	7.207	1.509	5.800	1.136	6.937
Glu2	-7.012	-1.002	4.007	6.009	1.336	5.051	1.044	6.095
Glu3	-6.885	-0.357	3.621	6.528	1.004	4.227	0.606	4.833
Glu4	-6.651	0.302	3.175	6.954	0.725	3.471	0.297	3.768
His1	-7.419	-1.159	4.289	6.260	1.469	5.474	1.185	6.659
His2	-7.052	-0.814	3.933	6.238	1.240	4.836	0.903	5.738
His3	-6.119	-0.476	3.298	5.643	0.963	3.928	0.631	4.559
His4	-5.801	-0.043	2.922	5.759	0.741	3.304	0.382	3.685
Lys1	-8.588	-1.072	4.830	7.516	1.552	5.988	1.159	7.147
Lys2	-7.273	-0.698	3.986	6.575	1.208	4.820	0.834	5.654
Lys3	-6.765	-0.666	3.715	6.099	1.132	4.502	0.787	5.289
Lys4	-6.719	0.158	3.280	6.877	0.782	3.635	0.354	3.989
	I	A	χ	η	ω	ω^-	ω^+	$\Delta\omega^\pm$
Arg1	7.491	1.029	4.260	6.462	1.404	5.342	1.082	6.425
Arg2	6.885	-0.307	3.289	7.193	0.752	3.598	0.309	3.907
Arg3	6.680	-0.363	3.158	7.043	0.708	3.436	0.277	3.713
Arg4	6.209	-0.637	2.786	6.847	0.567	2.955	0.169	3.124
Asp1	8.402	0.997	4.699	7.405	1.491	5.795	1.095	6.890
Asp2	6.756	0.756	3.756	6.000	1.176	4.605	0.848	5.453
Asp3	6.489	-0.137	3.176	6.626	0.761	3.524	0.348	3.873
Asp4	6.251	-0.573	2.839	6.823	0.591	3.027	0.188	3.215
Glu1	7.925	1.026	4.476	6.899	1.452	5.573	1.097	6.670
Glu2	6.508	0.944	3.726	5.564	1.248	4.706	0.980	5.686
Glu3	6.413	-0.371	3.021	6.785	0.673	3.280	0.259	3.538
Glu4	6.485	-0.796	2.845	7.281	0.556	2.989	0.144	3.133
His1	7.449	1.122	4.286	6.326	1.452	5.441	1.156	6.597
His2	6.910	0.879	3.895	6.031	1.258	4.840	0.945	5.784
His3	6.096	-0.270	2.913	6.366	0.667	3.188	0.275	3.462
His4	5.766	-0.328	2.719	6.093	0.607	2.954	0.235	3.188
Lys1	8.275	1.014	4.644	7.261	1.485	5.746	1.102	6.849
Lys2	6.869	-0.227	3.321	7.096	0.777	3.658	0.337	3.996
Lys3	6.435	-0.256	3.089	6.691	0.713	3.389	0.300	3.689
Lys4	6.626	-0.723	2.951	7.350	0.593	3.120	0.169	3.289

Table S3B. Descriptors J_I, J_A, J_{HL}, J_χ, J_η, J_ω, J_{D1}, J_{ω^-}, J_{ω^+}, $J_{\Delta\omega^\pm}$ and J_{D2} for the natural amino acids bearing a ionizable side-chain at different pHs calculated from the results of Table S3A

	J_I	J_A	J_{HL}	J_χ	J_η	J_ω	J_{D1}	J_{ω^-}	J_{ω^+}	$J_{\Delta\omega^\pm}$	J_{D2}
Arg1	0.06	0.05	0.08	0.06	0.01	0.03	0.07	0.10	0.04	0.14	0.17
Arg2	0.40	0.74	0.84	0.57	0.33	0.33	0.74	0.93	0.36	1.29	1.63
Arg3	0.10	0.75	0.75	0.42	0.65	0.29	0.83	0.76	0.34	1.10	1.37
Arg4	0.14	0.52	0.54	0.33	0.38	0.18	0.53	0.51	0.18	0.68	0.87
Asp1	0.19	0.11	0.22	0.15	0.08	0.08	0.19	0.24	0.09	0.33	0.42
Asp2	0.50	0.17	0.53	0.33	0.33	0.15	0.49	0.48	0.14	0.62	0.80
Asp3	0.49	0.56	0.75	0.53	0.07	0.28	0.60	0.83	0.30	1.13	1.43
Asp4	0.10	0.28	0.29	0.19	0.18	0.10	0.28	0.28	0.09	0.37	0.47
Glu1	0.34	0.03	0.34	0.19	0.31	0.06	0.37	0.23	0.04	0.27	0.35
Glu2	0.50	0.06	0.51	0.28	0.45	0.09	0.53	0.35	0.06	0.41	0.54
Glu3	0.47	0.73	0.87	0.60	0.26	0.33	0.73	0.95	0.35	1.29	1.64
Glu4	0.17	0.49	0.52	0.33	0.33	0.17	0.49	0.48	0.15	0.63	0.81
His1	0.03	0.04	0.05	0.00	0.07	0.02	0.07	0.03	0.03	0.06	0.08
His2	0.14	0.07	0.16	0.04	0.21	0.02	0.21	0.00	0.04	0.05	0.06
His3	0.02	0.75	0.75	0.38	0.72	0.30	0.87	0.74	0.36	1.10	1.37
His4	0.04	0.37	0.37	0.20	0.33	0.13	0.41	0.35	0.15	0.50	0.63
Lys1	0.31	0.06	0.32	0.19	0.25	0.07	0.32	0.24	0.06	0.30	0.39
Lys2	0.40	0.92	1.01	0.66	0.52	0.43	0.95	1.16	0.50	1.66	2.08
Lys3	0.33	0.92	0.98	0.63	0.59	0.42	0.96	1.11	0.49	1.60	2.01
Lys4	0.09	0.57	0.57	0.33	0.47	0.19	0.61	0.51	0.19	0.70	0.89
Average	0.24	0.41	0.52	0.32	0.33	0.18	0.51	0.51	0.20	0.71	0.90

Table S4A. HOMO and LUMO orbital energies (eV), ionization potential I and electron affinity A (eV), global electronegativity χ, chemical hardness η, global electrophilicity ω, electrodonating power ω^-, electroaccepting power ω^+ and net electrophilicity $\Delta\omega^{\pm}$ of natural amino acids bearing a ionizable side-chain at different pHs calculated with the N12SX density functional and the Def2TZVP basis set using water as solvent simulated with the SMD parametrization of the IEF-PCM model. The upper part of the table shows the results derived assuming the validity of the KID procedure and the lower part shows the results derived from the calculated vertical ΔSCF energies.

	HOMO	LUMO	χ_K	η_K	ω_K	ω_K^-	ω_K^+	$\Delta\omega_K^{\pm}$
Arg1	-7.405	-1.028	4.216	6.377	1.394	5.295	1.078	6.373
Arg2	-6.985	0.315	3.335	7.300	0.762	3.647	0.312	3.960
Arg3	-6.177	0.341	2.918	6.517	0.653	3.173	0.255	3.428
Arg4	-6.064	0.866	2.599	6.930	0.487	2.707	0.108	2.815
Asp1	-8.287	-1.017	4.652	7.270	1.489	5.758	1.105	6.863
Asp2	-6.865	-0.740	3.803	6.126	1.180	4.645	0.842	5.486
Asp3	-6.582	0.502	3.040	7.085	0.652	3.267	0.227	3.495
Asp4	-6.018	0.957	2.531	6.975	0.459	2.619	0.089	2.708
Glu1	-7.986	-1.070	4.528	6.916	1.482	5.660	1.133	6.793
Glu2	-6.605	-0.993	3.799	5.612	1.286	4.822	1.023	5.845
Glu3	-6.502	0.642	2.930	7.144	0.601	3.114	0.183	3.297
Glu4	-6.253	1.087	2.583	7.340	0.455	2.660	0.076	2.736
His1	-7.356	-1.131	4.244	6.226	1.446	5.403	1.160	6.563
His2	-6.825	-0.891	3.858	5.934	1.254	4.809	0.950	5.759
His3	-5.950	0.264	2.843	6.213	0.650	3.110	0.268	3.378
His4	-5.653	0.520	2.566	6.173	0.533	2.736	0.170	2.905
Lys1	-8.320	-1.003	4.662	7.317	1.485	5.758	1.097	6.855
Lys2	-6.988	0.479	3.255	7.467	0.709	3.513	0.258	3.771
Lys3	-6.382	0.521	2.931	6.904	0.622	3.141	0.210	3.351
Lys4	-6.333	1.058	2.638	7.392	0.471	2.722	0.084	2.806
	I	A	χ	η	ω	ω^-	ω^+	$\Delta\omega^{\pm}$
Arg1	7.399	1.085	4.242	6.313	1.425	5.366	1.124	6.489
Arg2	6.832	-0.282	3.275	7.114	0.754	3.590	0.315	3.905
Arg3	6.169	-0.307	2.931	6.476	0.663	3.197	0.266	3.463
Arg4	5.969	-0.663	2.653	6.633	0.531	2.802	0.149	2.951
Asp1	8.216	1.070	4.643	7.147	1.508	5.785	1.142	6.927
Asp2	6.672	0.806	3.739	5.866	1.192	4.620	0.880	5.500
Asp3	6.399	-0.214	3.092	6.613	0.723	3.405	0.313	3.719
Asp4	6.007	-0.729	2.639	6.737	0.517	2.774	0.135	2.910
Glu1	7.872	1.123	4.497	6.749	1.498	5.667	1.170	6.873
Glu2	6.517	1.047	3.782	5.470	1.307	4.847	1.065	5.913
Glu3	6.412	-0.506	2.953	6.917	0.630	3.169	0.216	3.386
Glu4	6.173	-0.806	2.684	6.979	0.516	2.810	0.126	2.936
His1	7.365	1.185	4.275	6.180	1.478	5.480	1.206	6.686
His2	6.754	0.969	3.861	5.785	1.289	4.869	1.008	5.877
His3	5.904	-0.179	2.862	6.083	0.673	3.158	0.296	3.454
His4	5.599	-0.414	2.592	6.014	0.559	2.790	0.197	2.987
Lys1	8.228	1.061	4.644	7.167	1.505	5.780	1.135	6.915
Lys2	6.824	-0.192	3.316	7.016	0.784	3.664	0.348	4.011
Lys3	6.402	-0.221	3.091	6.623	0.721	3.401	0.311	3.712
Lys4	6.285	-0.790	2.747	7.075	0.533	2.882	0.135	3.018

Table S4B. Descriptors J_I, J_A, J_{HL}, J_χ, J_η, J_ω, J_{D1}, J_{ω^-}, J_{ω^+}, $J_{\Delta\omega^\pm}$ and J_{D2} for the natural amino acids bearing a ionizable side-chain at different pHs calculated from the results of Table S4A

	J_I	J_A	J_{HL}	J_χ	J_η	J_ω	J_{D1}	J_{ω^-}	J_{ω^+}	$J_{\Delta\omega^\pm}$	J_{D2}
Arg1	0.01	0.06	0.06	0.03	0.06	0.03	0.08	0.07	0.05	0.12	0.14
Arg2	0.15	0.03	0.16	0.06	0.19	0.01	0.19	0.06	0.00	0.05	0.08
Arg3	0.01	0.03	0.03	0.01	0.04	0.01	0.04	0.02	0.01	0.04	0.04
Arg4	0.09	0.20	0.22	0.05	0.30	0.04	0.30	0.10	0.04	0.14	0.17
Asp1	0.07	0.05	0.09	0.01	0.12	0.02	0.12	0.03	0.04	0.06	0.08
Asp2	0.19	0.07	0.20	0.06	0.26	0.01	0.27	0.02	0.04	0.01	0.05
Asp3	0.18	0.29	0.34	0.05	0.47	0.07	0.48	0.14	0.09	0.22	0.28
Asp4	0.01	0.23	0.23	0.11	0.24	0.06	0.27	0.15	0.05	0.20	0.26
Glu1	0.11	0.05	0.13	0.03	0.17	0.02	0.17	0.01	0.04	0.04	0.06
Glu2	0.09	0.05	0.10	0.02	0.14	0.02	0.14	0.03	0.04	0.07	0.08
Glu3	0.09	0.14	0.16	0.02	0.23	0.03	0.23	0.06	0.03	0.09	0.11
Glu4	0.08	0.28	0.29	0.10	0.36	0.06	0.38	0.15	0.05	0.20	0.26
His1	0.01	0.05	0.05	0.03	0.05	0.03	0.06	0.08	0.05	0.12	0.15
His2	0.07	0.08	0.11	0.00	0.15	0.03	0.15	0.06	0.06	0.12	0.15
His3	0.05	0.08	0.10	0.02	0.13	0.02	0.13	0.05	0.03	0.08	0.09
His4	0.05	0.11	0.12	0.03	0.16	0.03	0.16	0.05	0.03	0.08	0.10
Lys1	0.09	0.06	0.11	0.02	0.15	0.02	0.15	0.02	0.04	0.06	0.08
Lys2	0.16	0.29	0.33	0.06	0.45	0.07	0.46	0.15	0.09	0.24	0.30
Lys3	0.02	0.30	0.30	0.16	0.28	0.10	0.34	0.26	0.10	0.36	0.46
Lys4	0.05	0.27	0.27	0.11	0.32	0.06	0.34	0.16	0.05	0.21	0.27
Average	0.08	0.14	0.17	0.05	0.21	0.04	0.22	0.08	0.05	0.13	0.16
